# Exploring the Links Between Cognitive Deficits and EEG Spectral Density in Schizophrenia

**DOI:** 10.1192/j.eurpsy.2025.2117

**Published:** 2025-08-26

**Authors:** N. Smaoui, D. Jardak, R. Feki, M. Bou Ali Maalej, I. Gassara, N. Charfi, J. Ben Thabet, M. Maalej, S. Omri, L. Zouari, L. Triki

**Affiliations:** 1 Psychiatry C, Hedi Chaker University Hospital; 2 Functional Explorations, Habib Bourguiba University Hospital, Sfax, Tunisia

## Abstract

**Introduction:**

The research revealed significant correlations between cognitive performance, assessed by psychometric scales, and variations in frequency bands in the electroencephalography (EEG), illustrating the link between electroencephalographic activity and cognitive functions in schizophrenic patients

**Objectives:**

Our study aimed to explore the relationship between the electroencephalographic spectral power of slow frequency bands (delta and theta) and cognitive functions in patients with schizophrenia by comparing them to healthy subjects.

**Methods:**

We conducted a cross-sectional, descriptive, and analytical study involving 15 schizophrenic patients and 15 healthy controls. The study was performed at the Psychiatry Department “C” outpatient unit at Hedi Chaker University Hospital in Sfax in Tunisia. We used the Arabic literary version of the Screen for Cognitive Impairment in Psychiatry (SCIP) scale to assess cognitive functions. Participants underwent a standard wakefulness EEG with eyes closed at the Functional Explorations Department of Habib Bourguiba Hospital in Sfax in Tunisia. Linear regression analysis was used to examine correlations between the total SCIP score and the absolute spectral density (ASD) values of EEG oscillations.

**Results:**

Linear regression analysis revealed a negative correlation between the total SCIP score and the delta wave ASD at T5 (left temporal) (r = -0.37; p = 0.025) and theta wave ASD at Fp2 (right prefrontal) (r = -0.131; p = 0.006). A positive correlation was found between theta wave ASD at F3 (left frontal) (r = 0.125; p = 0.02) and the total SCIP score. It revealed a negative correlation between the total SCIP score and the age of onset of schizophrenia (r = -0.647; p = 0.001).

**Conclusions:**

These results suggest that theta and delta power at rest, as measured by EEG, may serve as potential biomarkers for cognitive deficits in patients with schizophrenia. These findings could contribute to a better understanding of the neurophysiological basis of cognitive alterations associated with this condition.

**Disclosure of Interest:**

None Declared

